# The first rib as a method of adult age-at-death estimation in a modern South African sample

**DOI:** 10.1007/s00414-023-02978-3

**Published:** 2023-03-16

**Authors:** Nicolene Jooste, Maryna Steyn

**Affiliations:** 1 Department of Human Anatomy and Physiology, Faculty of Health Sciences, University of Johannesburg, Johannesburg, South Africa; 2 Human Variation and Identification Research Unit, School of Anatomical Sciences, Faculty of Health Sciences, University of the Witwatersrand, Johannesburg, South Africa

**Keywords:** First rib, Adult age-at-death, Forensic anthropology population data, Multiple regression analysis

## Abstract

An age-at-death estimation method using the first rib may be particularly advantageous as this rib is relatively easy to identify, not easily damaged postmortem, and associated with less mechanical stresses compared to other age indicators. Previously, mixed results have been achieved using the first rib to estimate age-at-death. This study aimed to develop and test an age-at-death estimation method using the first rib. An identified modern black South African sample of 260 skeletons were used to collect age-related data from the first rib. Multiple linear regression analysis equations were created from this data for male, female, and combined samples. When tested on a hold-out sample, equations generated mean inaccuracies of 7–13 years for point estimates. The 95% confidence intervals contained the true age in 11–33% of individuals depending on the equation used, but wider intervals generated using 95% prediction intervals contained true ages for 100% of individuals. Point estimate inaccuracies are comparable to other age-at-death estimation methods and may be useful if single indicator estimation is unavoidable in the case of missing or damaged bones. However, combined methods that use indicators from many areas of the skeleton are preferable and may reduce interval widths.

## Introduction

Adult age-at-death estimation from the skeleton is difficult because of inherent human variation as people age. Different statistical approaches and observer subjectivity add to the complexity of obtaining a reliable age estimate from the skeleton [1–5]. For this reason, researchers continue to investigate new biological indicators that may improve accuracy, precision, and repeatability individually or as part of a multivariate approach.

Kunos et al. [6] were the first to investigate the first rib, following on other studies that used other sternal rib ends as age indicators [2]. Their study documented age-related changes to the surface and texture appearance at the costochondral facet, tubercle, and head of the first rib. These documented changes not only described age-related progress in the adult skeleton but also incorporated skeletons between 1 and 75 years old. Subadult changes included ossification of secondary centers at the rib head and tubercle as well as metric changes to the rib length and costochondral surface.

These authors concluded that the overall age-at-death distribution pattern between observers (intra- and interobserver agreement) and also between the estimated and known age distributions were not statistically significantly different. As both these distribution patterns are calculated using the mean differences between observations, these reported averages could mask large individual differences. Kunos et al. note this by stating that individual specimen’s ages could markedly differ between observations and also between estimated and known ages [6]. Overall, they considered the first rib method especially useful compared to the popular fourth rib method [7], as the first rib is relatively easy to identify even in unarticulated skeletons or when damaged. They also considered the first rib exempt from the mechanical stresses common to the fourth rib. In conclusion, the authors suggested using the first rib method as part of a multifactorial approach.

A small independent validation (*n* = 29) of the Kunos et al. [6] first rib method concluded that the first rib method ranked particularly well for both accuracy (ranked third of nine) and bias (ranked first of nine) in the over 60-year category compared to contemporaneous methods from the fourth rib, pubic symphysis, auricular surface, and the cranial sutures [8]. Results from the first rib method for the under 60-year category were considered poor (ranked ninth—last place for both accuracy and bias). Another independent validation (*n* = 39) found that ages of only 55% of skeletons were correctly identified, and in contrast found that the over 60-year-old age group was misclassified most often, through underestimation [9]. Both validation studies highlighted drawbacks to the method such as subjective categorical descriptions, inadequate reference sample variation for application in more variable or dissimilar populations and the absence of age intervals [8, 9].

As advanced mathematical techniques have become increasingly popular to analyze known biological age-at-death indicators, the relationship between age and the first rib was also developed into a Bayesian analysis method [10]. DiGangi et al. [10] used a large sample (*n* = 470) of presumptively or positively identified males from single internment graves in Kosovo. They modified the Kunos et al. [6] method significantly to produce eleven variables with between three and five age-related categories representing each. Ultimately, only two variables were chosen for the final technique due to high correlations between individual traits. In contrast to previous findings, DiGangi et al. [10] found their method to be equally applicable to younger- and older-aged skeletons.

Merrit [11] noticed that reference sample distributions often differed notably between original and revised methods, which could influence results. This is also true for the first rib method, where the Kunos et al. [6] method was developed using 74 first ribs (and tested using 182 first ribs) but has approximately equal representation for each decade and sex, while DiGangi et al. [10] used a large (*n* = 470) sample of convenience that contained only males. Thus, Merrit [11] used a small sample (*n* = 20) of male individuals from European ancestry to compare the performance of, among other methods, the Kunos et al. [6] and DiGangi et al. [10] methods [11]. Unfortunately, even though this study highlighted the potential impact of reference sample distribution on the performance of methods, the test sample was not equally distributed between decades and contains many older individuals. Overall, the Kunos et al. [6] point estimates were observed to outperform the DiGangi et al. [10] point estimates, with the latter method statistically significantly underestimating ages compared to the former. For the over 60-year category, Merrit [11] not only found the Kunos et al. [6] method to be particularly accurate as per previous validation tests [8], but the most accurate compared to other contemporaneous methods (i.e., İşcan et al. [12], Lovejoy et al. [13], Todd [14], and Brooks and Suchey [15]). In contrast, the DiGangi et al. [10] method was the least accurate in the over 60 age group compared to contemporaneous methods (i.e., Hartnett [16, 17], Passalacqua [18], Buckberry and Chamberlain [19], and Rougé-Maillart et al. [20]). Overall, the DiGangi et al. [10] method was found to correctly assign individuals to age phases (i.e., 20–29, 40–59, 60 + years) more often (18/20) compared to the Kunos et al. [6] method (15/20), especially for the 20–39 year category. The Kunos et al. [6] method was considered the most reproducible of all methods tested, in stark contrast to previous validation conclusions. DiGangi et al. [10] produced the lowest intra-observer methods from all assessed. It is worth noting that the Kunos et al. [6] method’s reproducibility was assessed using an interclass correlation coefficient compared to weighted Cohen’s kappa tests for the remainder of the methods, due to the different result structure. Merrit [11] very astutely concludes that even though in most cases the newer/revised methods outperformed the originals, 95% confidence intervals generated by these methods have become so large that it is almost impossible to incorrectly estimate the age-of-death.

One of the current debates centers around the use of statistics when more than one variable is available, with the most commonly used frequency statistics and Bayesian methods each having its own supporters [21]. A number of studies have indicated that Bayesian analysis does not outperform more simplistic mathematical methods [22–24]. It is also difficult to use without appropriate software programs, making it less user-friendly.

This study aimed to test the utility of the first rib as age-at-death indicator for a modern black South African population and to produce a population-specific regression analysis method for use in forensic analyses. The regression equations were also evaluated using a hold-out test sample.

## Materials and methods

The right first ribs of 260 Black South African skeletons from the Raymond A Dart Collection of Human Skeletons were assessed for adult age-at-death information. The Raymond A Dart Collection of Human Skeletons in the School of Anatomical Sciences at the University of the Witwatersrand houses over 2500 remains with documented demographic data and grows annually with donated and unclaimed remains [25]. To ensure geographical and temporal relevance, skeletons were selected from the most recent additions as far as possible (date of birth: 1851–1965; date of death: 1926–1995). The sample was approximately equally distributed for each decade of adulthood (20–80 + years) as well as for both sexes.

After a pilot study was done, it was found that the features as described by DiGangi et al. [5] were very difficult to score repeatably and the distinctions between categories were somewhat ambiguous. Some of the described features were not present in the current sample. A few modifications were thus made to simplify the scoring criteria for the rib head (RH), tubercle facet (TF), and sternal end (CF1, CF2, and CF3), particularly with combining some of the previously described phases. These simplified scoring criteria are shown in Table 1. All specimens were scored blindly, without the observer knowing the age. Score observations were repeated on 28 ribs from the reference sample by the original and a second researcher in order to assess repeatability of the modified method. Repeatability of the scoring technique was assessed for single observers and multiple observers using a Cohen’s kappa analysis.

**Table 1:**
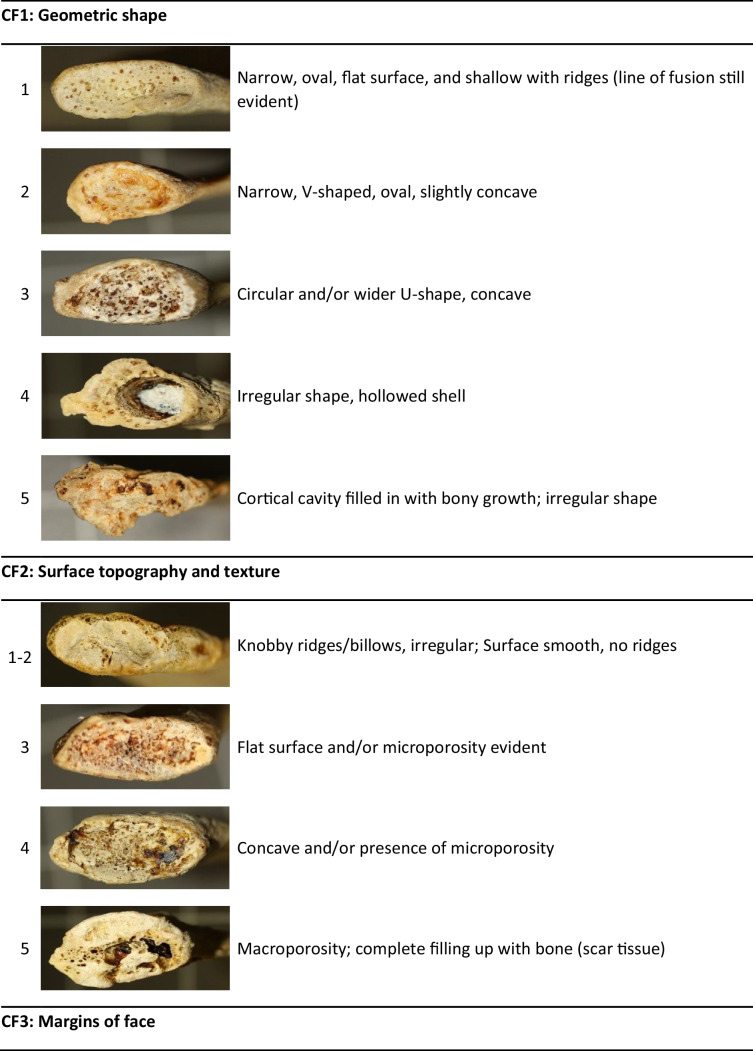

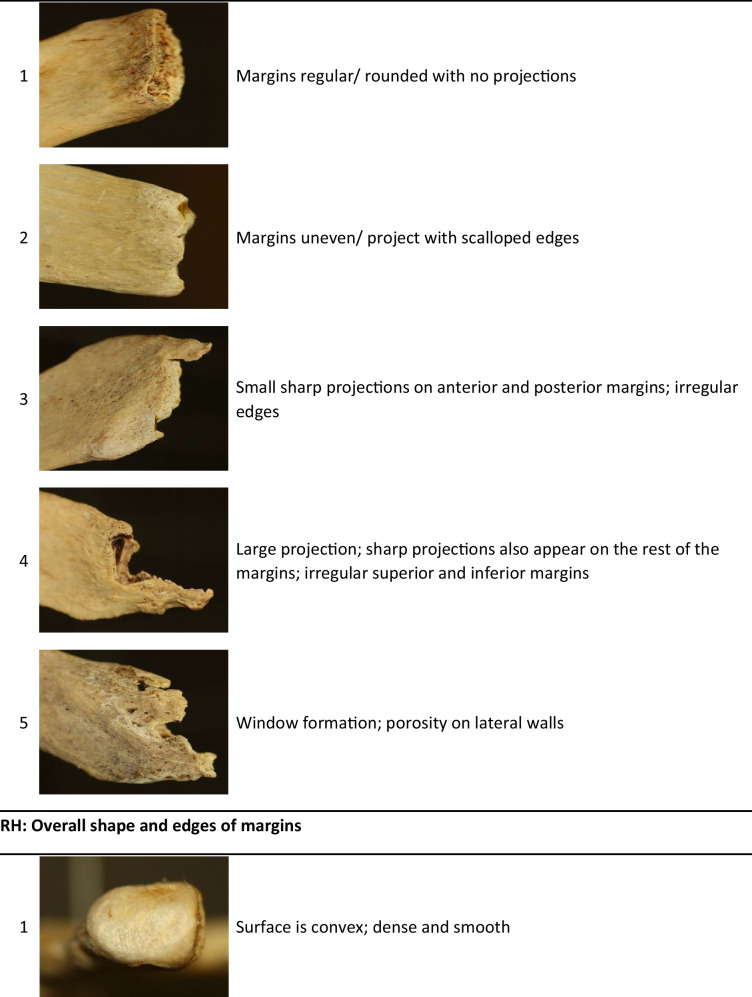

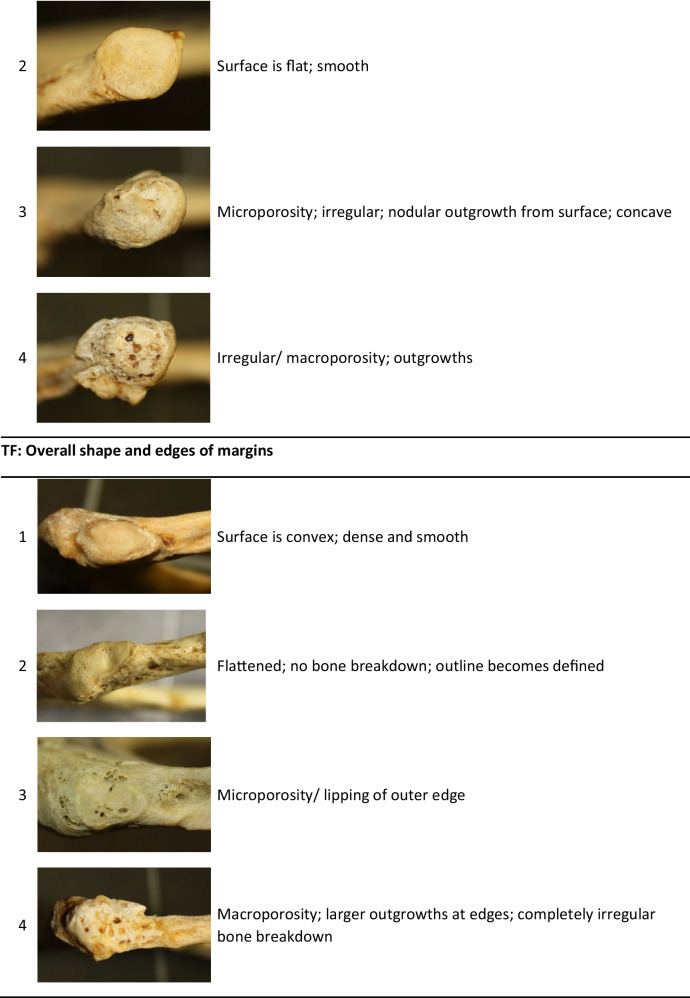
Descriptions of age-related features on Rib 1 adapted from DiGangi et al. [1] (photos by TMR Houlton). CF = costal face, RH = rib head, TF = tubercle facet

Statistics and graphs were analyzed using IBM SPSS Statistics 25 software package [26]. Boxplots were constructed for each possible category within each of the five features to assess the age-related data represented by each feature. Outliers were investigated, but only removed if records indicated pathological changes, post-mortem damage or possible erroneous documented ages. Category 1 of CF2 was represented by less than 2% of the sample and was combined with Category 2 to maximize the predictive data of these features.

Score distributions per category for males and females appeared dissimilar when visually inspected as boxplots. A Mann–Whitney *U* test was thus used to further compared the statistically significant differences in score distribution between the males and females.

Multiple linear regressions were calculated for the combined sample and the separate male and female subgroups. Features were assessed for significance of contribution to construct the most appropriate equation using a manual hierarchical technique.

Validity of the regression method was analyzed using 25 out-of-sample right first ribs from the Pretoria Bone Collection [27], which is also in the Gauteng region of South Africa. The validation sample was chosen from the most recent skeletons of African ancestry, from both sexes, to ensure applicability to the reference sample and the current living population (date of birth: 1910–1974; date of death: 1985–2011). An age-at-death point estimate and a 95% confidence interval were calculated for each test subject. From these age-at-death estimates inaccuracy, bias and interval widths and accuracies were calculated.

## Results

**Fig. 1:**
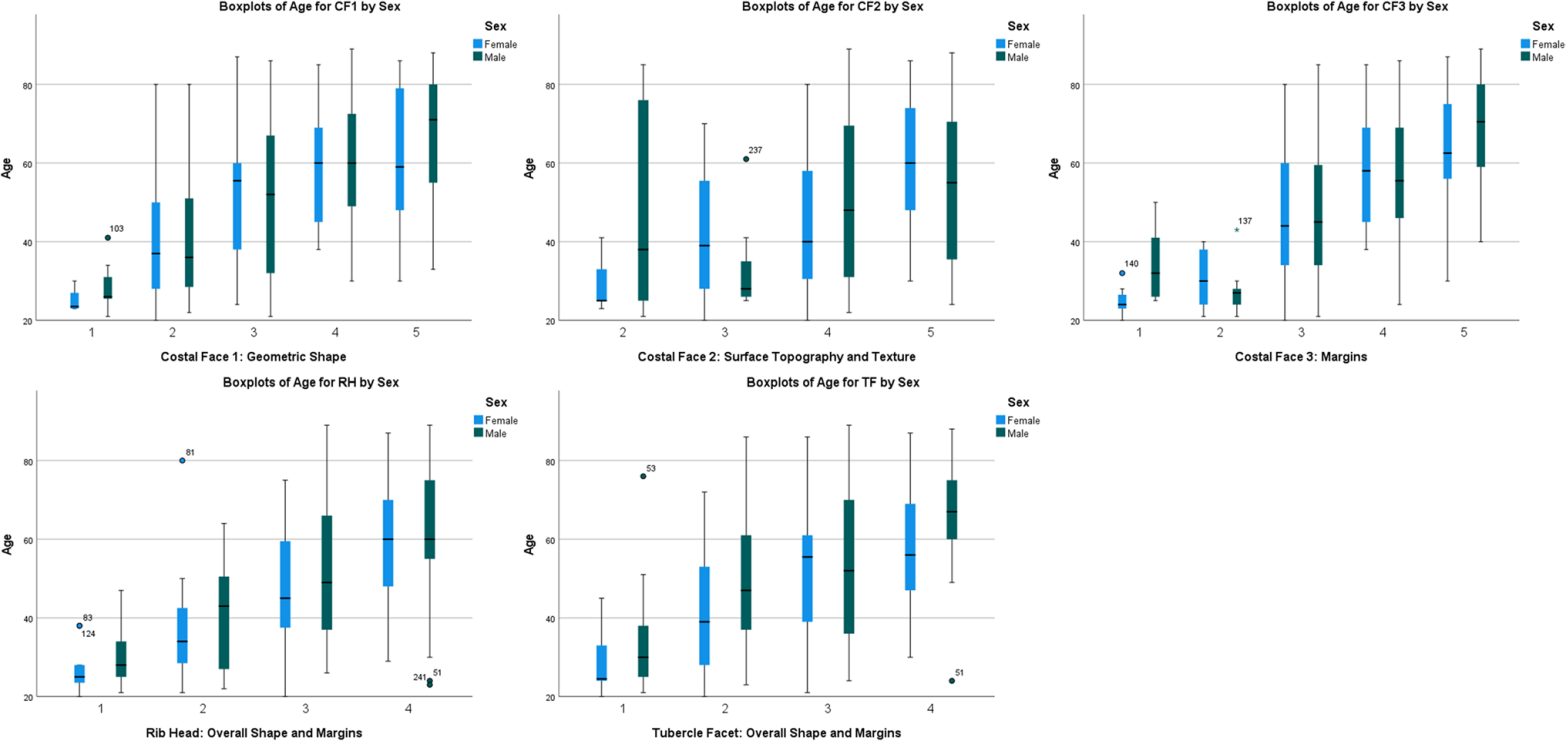
Boxplots showing the sex-specific age distribution by category for the five age indicators of rib 1

Boxplots of each feature (Fig. 1) indicated some increase in age with each progressive category when considering the medians. Unfortunately, higher numbered categories were represented by large age ranges that overlapped considerably. Large age ranges represent individual variation in rate of progression and will ultimately lead to less precise predictions using these features.

A Mann–Whitney *U* test indicated that the scores for CF1 (*U* = 6690, *z* =  − 2.979, *p* = 0.003), CF2 (*U* = 2104, *z* =  − 3.484, *p* < 0.001), and CF3 (*U* = 7012, *z* =  − 2.032, *p* = 0.042) were statistically significantly higher for males compared to females. This significant difference might be indicative of unique patterns of aging due to lifestyle differences and suggested that functions of prediction should be adapted to consider sex.

Initial results for multiple linear regression containing all features (CF1, CF2, CF3, RH, and TF) showed a 69% correlation with age using the combined sample and 64% and 75% for the female and male subgroups, respectively. However, all five features did not contribute statistically significantly to the multifactorial regression equation. Thus, the features with a low impact on the equation were manually excluded to produce the final equations in Table 2.

**Table 2:** Multiple regression equations for pooled, male, and female samples

Pooled sample (*N* = 230)	
	Age = 7.014 + (6.388 × CF3) + (4.050 × RH) + (3.286 × TF)
	*R* = 0.660; *R*^2^ = 0.436; adjusted *R*^2^ = 0.428; *F*(3,226) = 58.133, *p* < 0.001; SEE = 13.910
Male sample (*N* = 123)	
	Age = 6.971 + (6.381 × CF3) + (3.531 × RH) + (4.354 × TF)
	*R* = 0.684; *R*^2^ = 0.468; adjusted *R*^2^ = 0.454; *F*(3,119) = 34.873, *p* < 0.001; SEE = 14.196
Female sample (*N* = 107)	
	Age = 7.971 + (5.950 × CF3) + (4.812 × RH) + (2.115 × TF)
	*R* = 0.625; *R*^2^ = 0.390; adjusted *R*^2^ = 0.373; *F*(3,103) = 21.986, *p* < 0.001; SEE = 13.647

CF1 and CF2 did not contribute to any of the equations—combined or divided by sex. In addition to CF1 and CF2, the coefficient for TF did not contribute statistically significantly to the female equation but was retained as it contributes practically to the method as an additional data point. Highest correlations with age were achieved for the male equation (*r* = 68%), but using the combined sex equation produced higher correlations than the female equation (*r* = 66% compared to *r* = 63%). Only between 39 and 47% (*r*^2^) of age variability is accounted for by the independent variables (Table 2), with the female equation again having the lowest *r*^2^ value. Thus, less than 50% of change is accounted for by age and is more likely explained by other factors, especially for the female subgroup.

Mean absolute error for out-of-sample age-at-death point estimates indicated a mean inaccuracy of between 7 and 13 years (Table 3). On average, the age-at-death point estimate for the combined and female samples were consistently overestimated as indicated by the bias values in Table 3. The male sample presented a mean bias of zero. The 95% confidence intervals accurately contained the true ages for between 11 and 33% of individuals, which most likely indicates that the mean age intervals of 8 to 13 years were too narrow. However, 95% prediction intervals contain 100% of the true ages within the estimated intervals, with an increase in mean interval width to between 56 and 60 years. The wider, but more accurate, results for the 95% prediction interval (which estimates an interval for an individual observation) compared to the 95% confidence interval (which reports the likely range associated with the wider population) is expected.

**Table 3:** Validation of regression equations for pooled, male, and female samples

	Mean inaccuracy (max) in years	Mean bias in years	95% CI: mean interval width (max) in years	95% PI: mean interval width (max) in years	95% CI: N accurate (% accurate)	95% PI: N accurate (% accurate)
Pooled sample (*N* = 21)	9 (18)	-3	8 (15)	55 (57)	4 (19)	21 (100)
Male sample (*N* = 12)	7 (18)	0	10 (20)	57 (60)	4 (33)	12 (100)
Female sample (*N* = 9)	13 (19)	-8	13 (15)	56 (56)	1 (11)	9 (100)

The Cohen’s kappa statistics (Table 4) confirms the subjective nature of the scoring procedure. While kappa values for intraobserver agreement were all above 0.68, the interobserver values were lower. However, using the Landis and Koch [28] measurement strength, the kappa values for interobserver agreement are still considered fair and intraobserver substantial for the three variables included in the final equations (Table 4).

**Table 4:** Cohen’s kappa statistic for strength of inter- and intraobserver agreement

	Interobserver agreement			Intraobserver agreement		
	Cohen’s kappa (K)	*p* value	Measure of strength (Landis & Koch, 1977)	Cohen’s kappa (K)	*p* value	Measure of strength (Landis & Koch, 1977)
CF1	0.634	< 0.005	Substantial	0.863	< 0.005	Almost perfect
CF2	0.452	< 0.005	Moderate	0.840	< 0.005	Almost perfect
CF3	0.283	0.002	Fair	0.759	< 0.005	Substantial
RH	0.356	< 0.005	Fair	0.693	< 0.005	Substantial
TF	0.276	0.006	Fair	0.689	< 0.005	Substantial

## Discussion

In this paper, we adapted and condensed the scoring criteria for the first rib, as published by DiGangi et al. [10]. It was found that the initial criteria were overly detailed, and not all these features could be observed in the current sample. A sample of 260 first ribs of black males and females were used to construct regression equations, which were then tested with a sample of 21 black individuals from a different skeletal collection.

From the box plots (Fig. 1), it was clear that much overlap exists between categories. The relatively low contribution to age-at-death from the features in the regression equations (*r*^2^ values, Table 2), suggests that many factors contributing to structural changes over time are still unaccounted for. The categorical changes occurring at the costal face—CF1 (geometric shape) and CF2 (surface topography and texture)—did not contribute to the predictive equations in this sample population. Such factors could be environmental such as health and socioeconomic status or individual variation representing genetic variation. One such factor, documented by Merrit [29], is body mass index (BMI), related to body size and stature. According to Merrit’s [29. ] study, the Kunos et al. [6] method was the most reliable age-at-death estimation method for all BMI groups, body sizes, and statures and performed especially well in the underweight, light body size, and short stature categories [29]. The DiGangi et al. [10] method was the least reliable with more than a 10-year inaccuracy, which resulted in consistent underestimated ages [29]. The current paper could not draw conclusions based on BMI, body size, or antemortem stature, as this information was not available.

One factor that clearly influences the relationship between the structural changes of the first rib and age, in the current study, is sex. This could be an unintentional effect introduced by the category descriptions of DiGangi et al. [10], which were adapted for this current study and originally based on an all-male sample. This factor was accounted for by producing equations that represent the males and females from the sample separately. Sex-specific equations for the male subgroup also increased the performance of the equation (increase in *r*^2^ and accuracy). Unfortunately, the equation for the female subgroup performs particularly poorly (low *r*^2^ and accuracy). One explanation for the poor accuracy of the female group could be the underlying relationships between skeletal aging and female sex hormones or pregnancy. Any such female-specific confounders could be poorly accounted for due to the original all-male sample used for category description development. Thus, revisiting category descriptions for female samples may be advisable.

Although some of the out-of-sample validation results were disappointing, many of these results are comparable to previous studies. Not only are the mean inaccuracies for the point estimates of the current study (13, 7, and 9 years for female, male, and pooled, Table 3) comparable to the validation study performed by Kurki [8] (inaccuracy of 10.4 years), but also with other age estimation methods tested on a South African sample. Jones et al. [30] achieved mean inaccuracy values of 10.5, 10.7, and 10.6 years for the pubic symphysis and 15.9, 11.8, and 13.8 years for the auricular surface in a female, male, and pooled sample, respectively. When considering the percentage of individuals correctly aged by the calculated intervals, the wider prediction intervals produced 100% accuracy. These accuracies outperform those reported for other studies: 51 and 60% for males and females in a test of the first rib by Schmitt and Murail [9], and 36 and 35% for males and females using the fourth rib in a South African sample [31]. However, an associated increase in interval width produces impractical average widths of 56, 57, and 55 years (Table 3) for females, males, and pooled sample, respectively. These average interval widths may be reduced by using a multifactorial method. A multifactorial method developed in a different study, using a South African sample [24], achieved interval accuracies of 93–100% with slightly reduced average interval widths of 46, 53, and 52 years for females, males, and pooled samples. Using a multifactorial method may also improve results for a female sample, as alternative data will supplement the information from the first rib which does not seem to be well-suited to a female sample.

In conclusion, the first rib contains some age-related information that can be used to make predictions related to age-at-death but should ideally be used in combination with other features from the skeleton. It performed better in males than in females. This study once again demonstrates the difficulties with adult age estimation, with narrow, accurate estimates using macroscopic features still remaining elusive.

## Data Availability

All data can be made available upon request.
